# Immediate Implant Placement in the Mandibular Molar Septum Using Osseodensification: A Case Report with 4-Year Follow-Up

**DOI:** 10.3390/reports9030278

**Published:** 2026-08-21

**Authors:** Márcio de Carvalho Formiga, Jucielly Santos Lesnik de Souza, Gustavo dos Santos Coura, Sérgio Alexandre Gehrke

**Affiliations:** 1 Department of Implantology, Vale do Itajaí University (UNIVALI), Km 207, BR 101, São José 88103-800, Brazil; 2 Department of Pharmaceutical Science, School of Health Sciences, Vale do Itajai University (UNIVALI), Itajai 88302-901, Brazil

**Keywords:** dental implants, osseodensification, immediate implant placement, primary stability

## Abstract

**Background and Clinical Significance**: Immediate implant placement in mandibular molars represents a clinical challenge due to lower trabecular bone density and anatomical limitations of the interradicular septum. Osseodensification has been proposed as an alternative to improve primary stability in sites with low bone quality. **Case Presentation**: This report describes the case of a 54-year-old female patient with a mesial root fracture in the lower right first molar, treated by atraumatic extraction followed by immediate implant placement using a densifying drilling protocol. After careful root removal, the interradicular septum was expanded using Versah burs at 1200 rpm, allowing placement of a 4.0 × 7 mm Implacil Duecone implant with a final insertion torque of 25 Ncm. The residual gap was filled with a xenogeneic bone substitute and covered with a dense polytetrafluorethylene membrane for alveolar preservation. Healing progressed uneventfully and, after four months, adequate bone formation was observed, enabling prosthetic rehabilitation with a single ceramic crown. After 4 postoperative years we could find peri-implant tissue stability. **Conclusions**: The clinical outcome supports current evidence indicating that osseodensification may enhance initial stability and predictability of immediate implants placed in posterior mandibular regions.

## 1. Introduction and Clinical Significance

Immediate dental implant placement has been employed to reduce total treatment time, preserve alveolar architecture, and facilitate proper three-dimensional implant positioning. However, the predictability of this approach is directly linked to achieving satisfactory primary stability, particularly in posterior regions, where trabecular bone density is often low, and initial mechanical resistance may be limited [1,2].

In the mandibular molar region, immediate implant placement is considered technically challenging due to the wide diameter of the post-extraction socket, lower bone quality, variable inter-radicular septum width, and anatomical limitations imposed by the proximity of the inferior alveolar canal [3,4]. Under these circumstances, conventional osteotomy preparation can result in excessive removal of the remaining bone and a reduction in the implant–bone contact area, thereby increasing the risk of micromotion during the initial healing phase and compromising the predictability of immediate implants [2,5].

Primary stability plays a pivotal role in this context, as it represents the implant’s initial mechanical anchorage until biological stability is established. Evidence indicates that factors such as bone quality, surgical site preparation technique, and implant macro-geometry directly influence this initial phase [1,6]. When primary stability is insufficient, the likelihood of micromotion exceeding physiological limits increases, favoring fibrous tissue formation at the implant–bone interface and impairing osseointegration [2,5].

As an alternative to the limitations associated with conventional preparation, the osseodensification technique has been proposed as a biomechanical approach capable of preserving and compacting autogenous bone during drilling [3,7]. Experimental studies demonstrate that this protocol can result in increased peri-implant bone density and allow for controlled expansion of the alveolar ridge, promoting greater implant–bone contact, particularly in areas of low bone density [7,8].

Clinical evidence indicates that osseodensification can yield higher insertion torque values and more consistent primary stability than conventional osteotomy, including in posterior regions and with immediate implant placement [9,10]. Furthermore, the formation of compacted bone fragments during drilling acts as a nucleating agent, potentially accelerating osteogenesis and contributing to the maintenance of secondary stability throughout the healing process [11,12].

Clinical trials, comparative studies, and retrospective analyses have demonstrated reduced marginal bone loss, long-term stability maintenance, and favorable biomechanical behavior in implants placed using osseodensification, including immediate implants and those in posterior regions [13,14,15]. Given this body of evidence, this case report aims to describe the clinical application of the osseodensification technique for immediate implant placement in a mandibular molar site.

## 2. Case Presentation

A 54-year-old female patient presented to the clinic in October 2020 complaining of pain in the lower right first molar during clenching. Clinical evaluation revealed a positive vertical percussion test. Cone-beam computed tomography (CBCT) imaging showed a crack in the mesial root of the tooth, compromising its prognosis and indicating the need for extraction.

**Figure 1:**
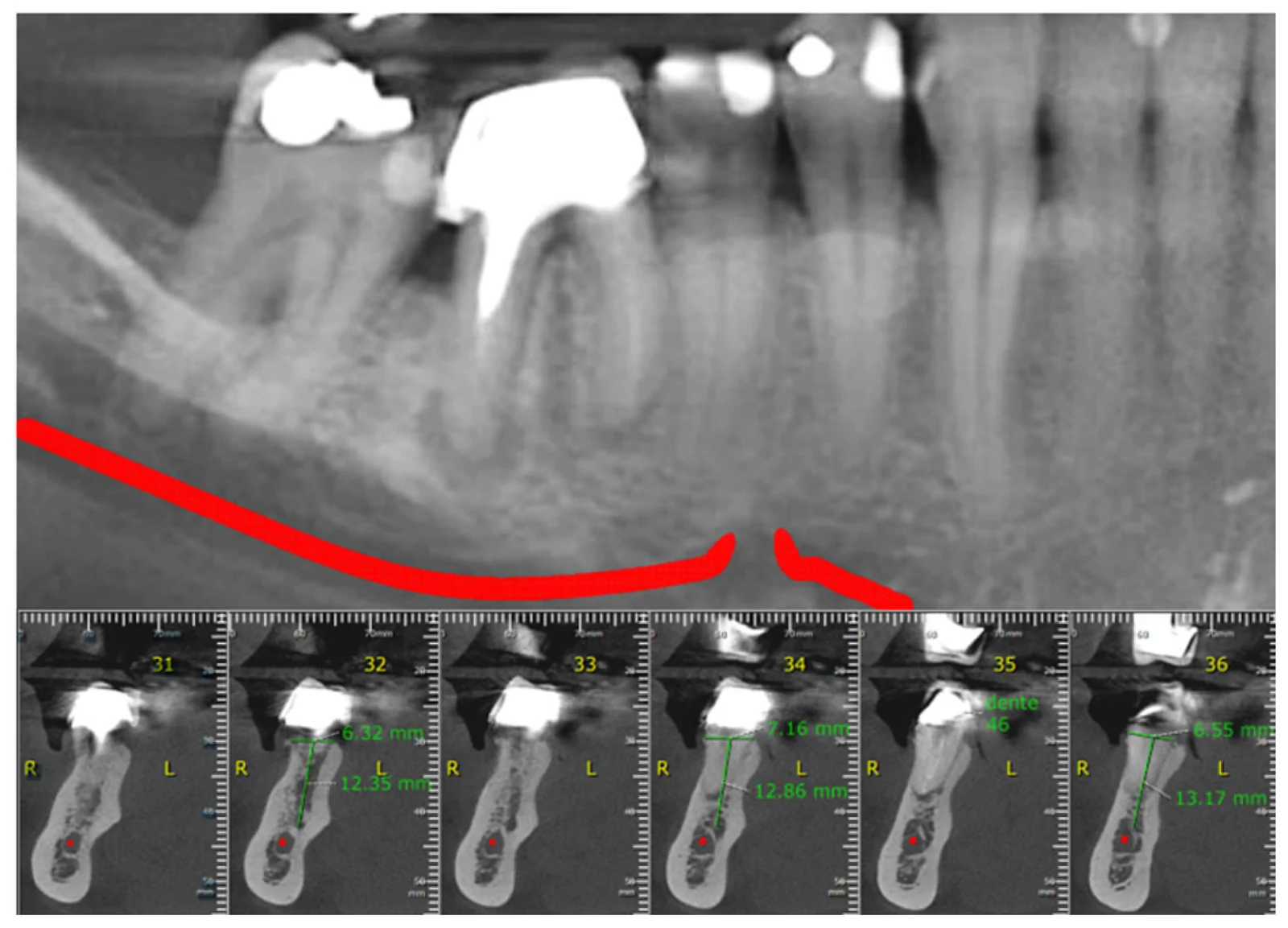
Baseline cone-beam computed tomography.

Given the lack of sufficient apical bone for conventional implant anchorage and the presence of a remaining inter-radicular septum, the treatment plan involved immediate implant placement into the septum, provided its integrity could be preserved during the surgical procedure. After a detailed explanation regarding the treatment plan, risks, benefits, and therapeutic alternatives, the patient signed the Informed Consent Form, authorizing the proposed procedure as well as the use of images and clinical data for scientific and academic purposes (Figure 1). Despite the very thin width of keratinized tissue around the molar, the patient did not consent to the removal of a free gingival graft from the palate to increase the band of keratinized tissue around the implant during the second surgery.

Preoperative medication consisted of 500 mg of amoxicillin every eight hours for seven days, starting two days before the surgical procedure, and 4 mg of dexamethasone, administered as a two-tablet dose one hour before surgery, for swelling control. Before the procedure, the patient performed a mouth rinse with 0.12% chlorhexidine digluconate for intraoral antisepsis, and extra oral antisepsis was performed using 2% chlorhexidine. Local anesthesia was achieved via infiltration using 4% articaine combined with 1:100,000 epinephrine.

**Figure 2:**
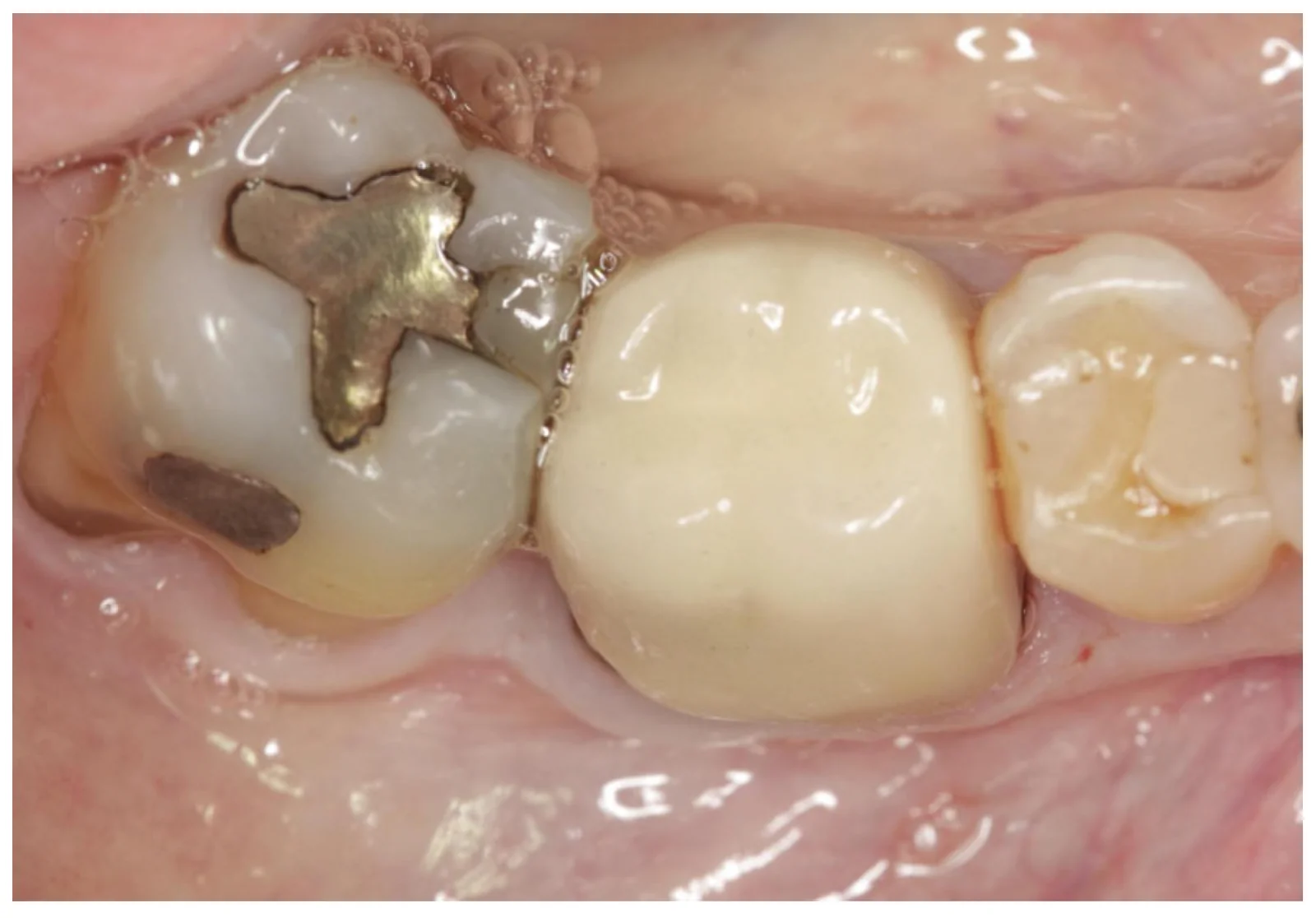
Prosthetic crown.

**Figure 3:**
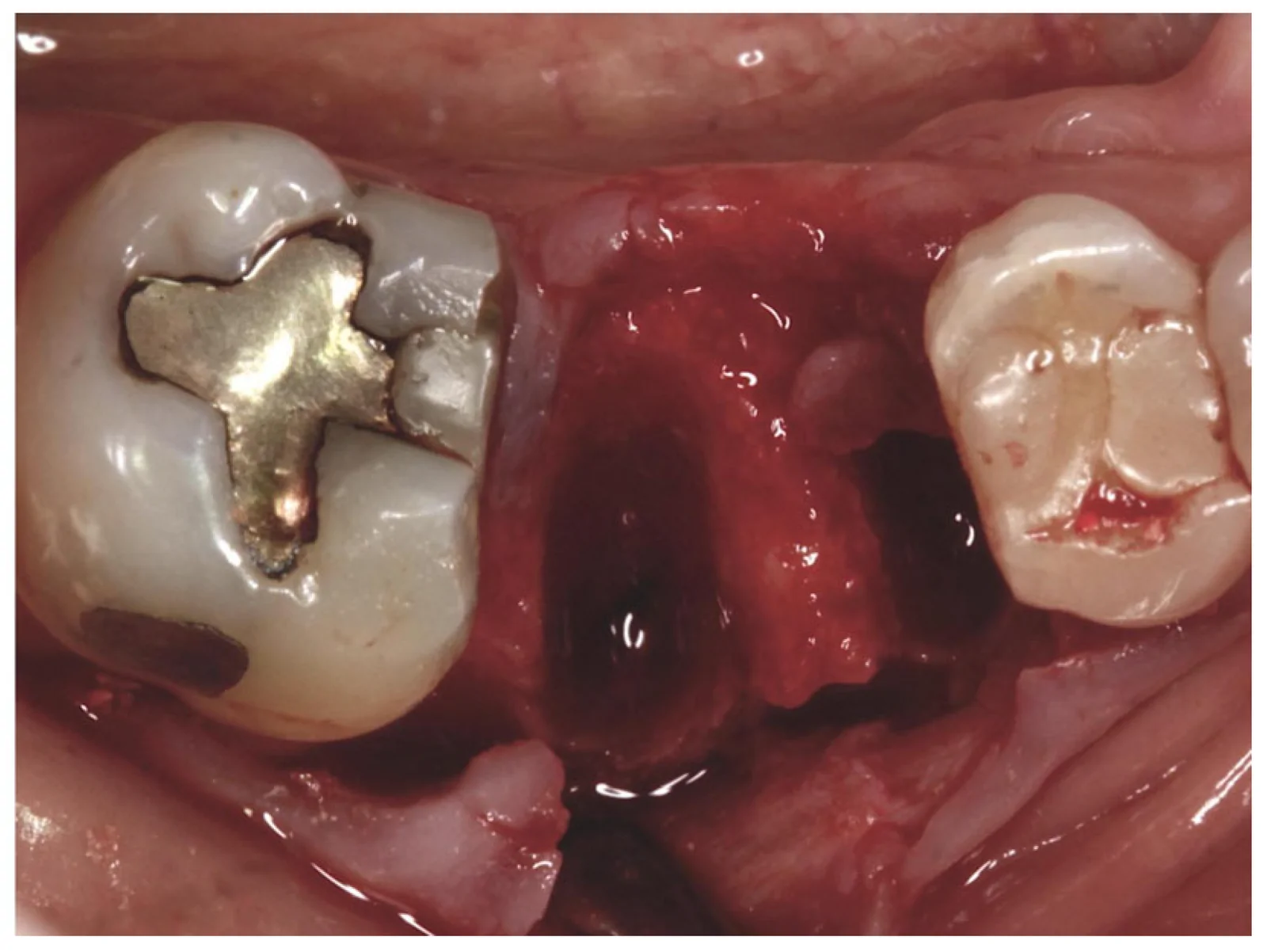
Appearance of the socket after extraction, showing loss of the buccal bone plate and preservation of the inter-radicular septum.

Initially, the prosthetic crown was removed with the help of a prosthesis remover, followed by odonto-section with a 701 high-speed bur. Then a periotome was used to cut the periodontal ligament and to start to luxate the separated roots. After this, we performed atraumatic root extraction using forceps number 151, aiming to preserve the interradicular septum as much as possible (Figure 2). Following the extraction, significant dehiscence in the buccal bone plate was observed; however, the central septum remained intact, allowing the proposed treatment plan to proceed (Figure 3).

**Figure 4:**
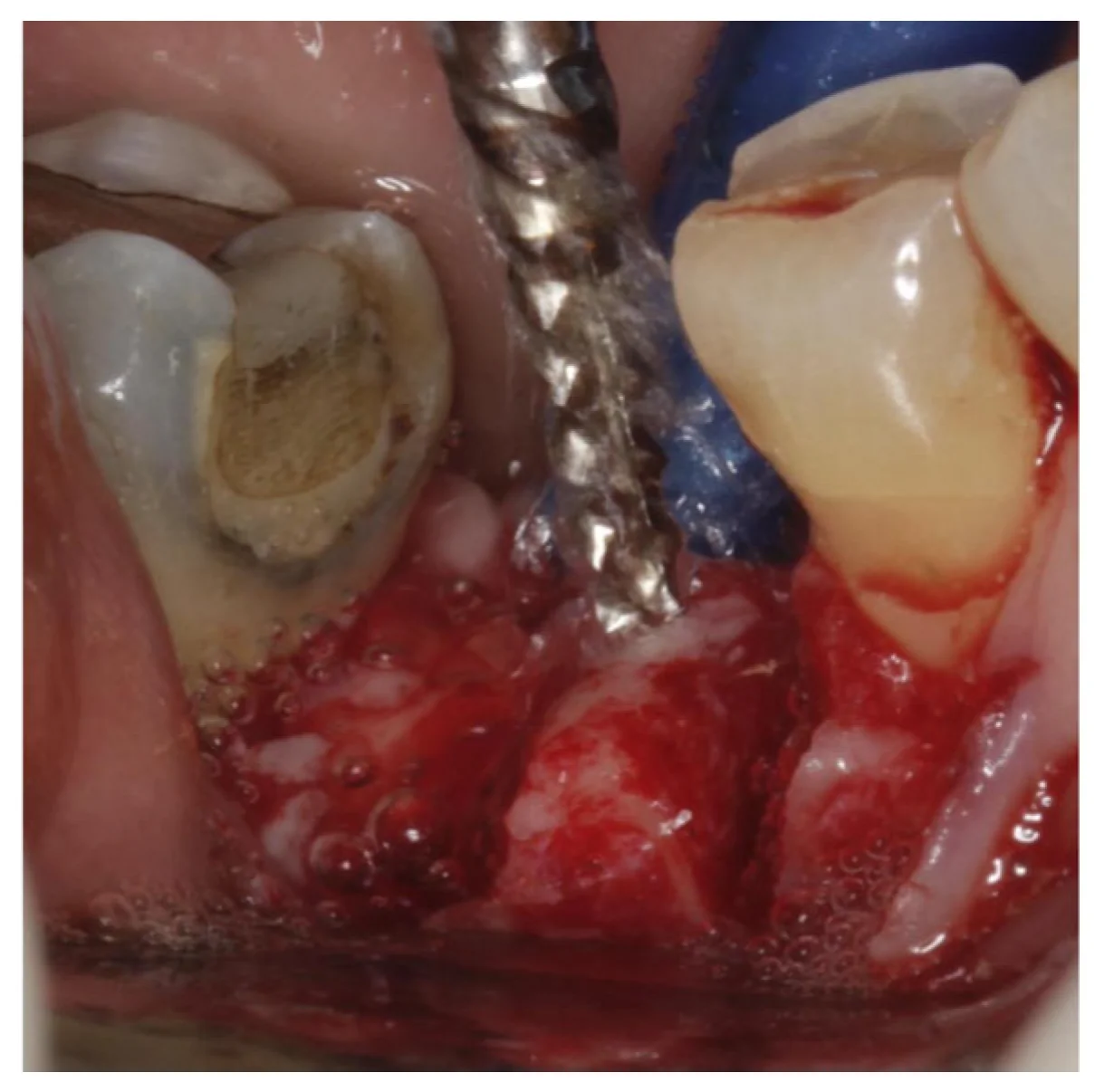
Initial implant site preparation in the center of the inter-radicular septum using a super-sharp drill.

**Figure 5:**
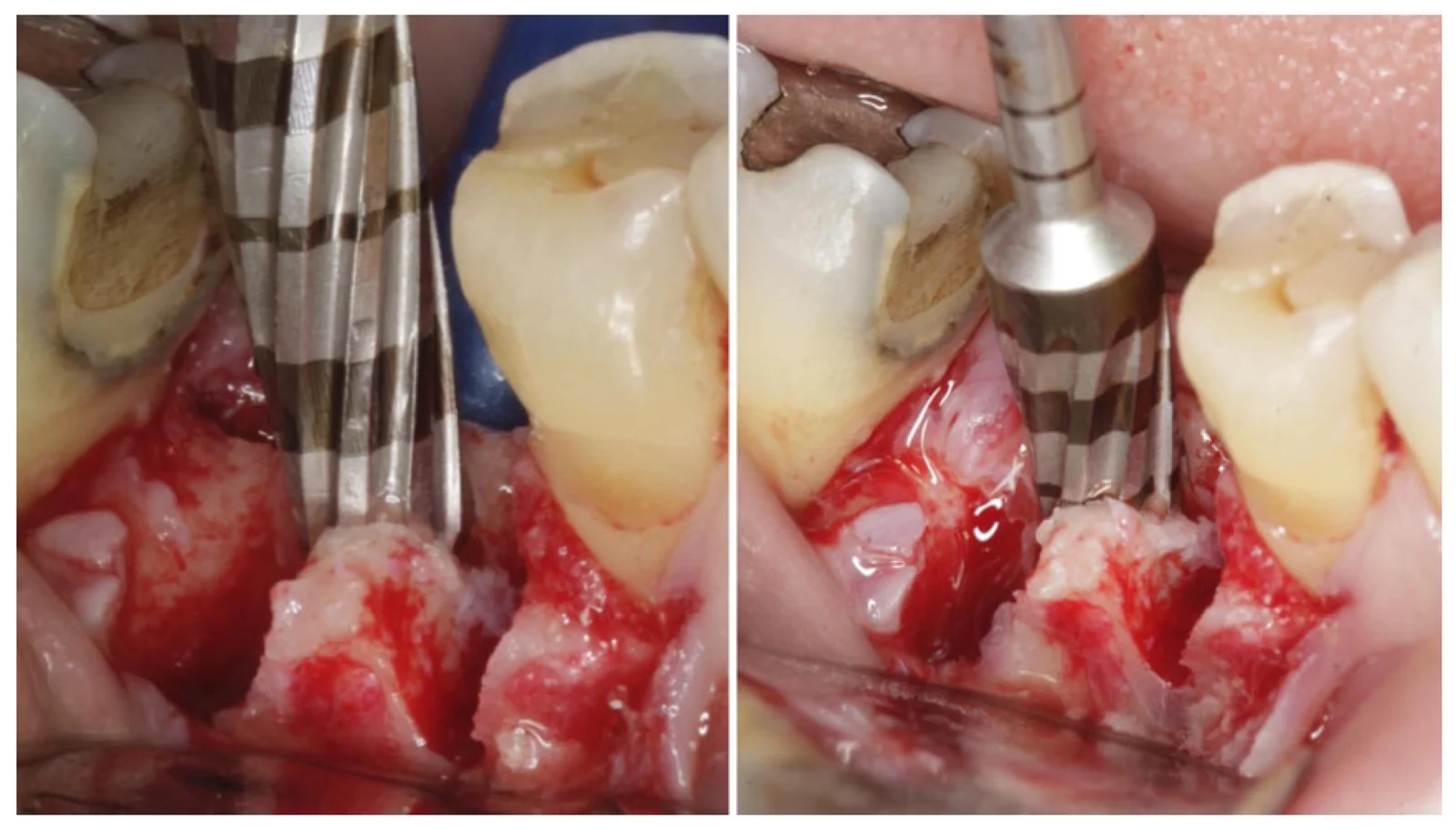
Instrumentation of the inter-radicular septum using Versah system drills following the osseodensification protocol.

**Figure 6:**
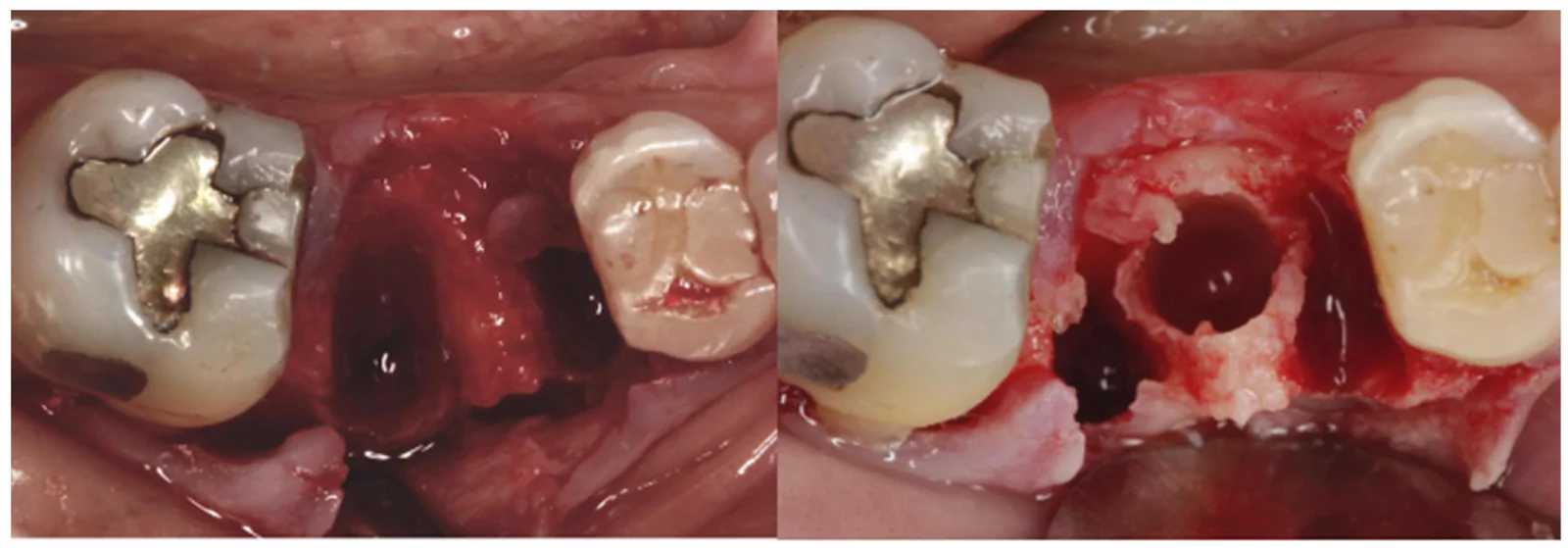
Clinical aspect before and after controlled expansion of the inter-radicular septum.

Initial preparation of the implant site was performed in the center of the inter-radicular septum using a super-sharp drill from the Implacil system to establish a stable guide axis for subsequent instrumentation (Figure 4). Next, osteotomy was performed in a counterclockwise direction using Versah system drills (Versal LLC, Jackson, MI, USA), following the osseodensification protocol (burs 2.0, 2.3, 3.0, 3.3, and final 4.0) at 1200 rpm with a surgical motor and a 20:1 contra-angle handpiece (NSK). Instrumentation was carried out progressively, promoting controlled expansion of the inter-radicular septum (Figure 5 and Figure 6).

**Figure 7:**
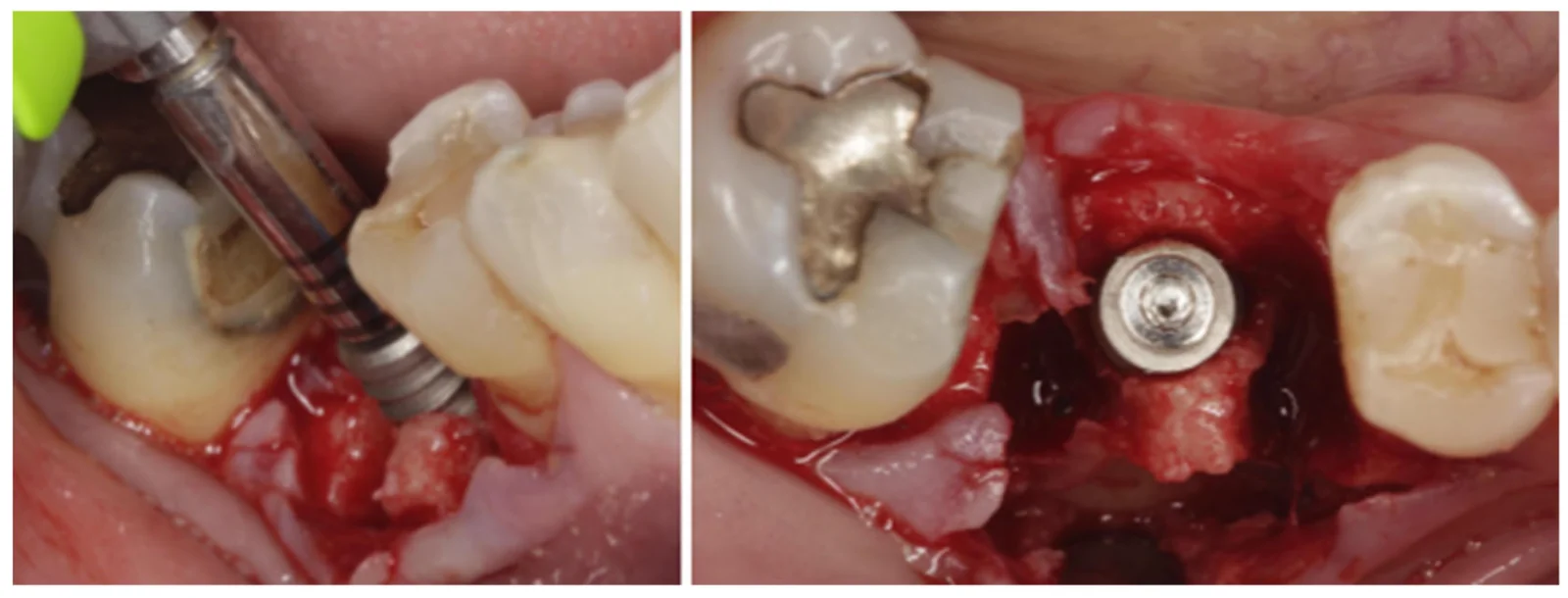
Placement of the Implacil Duecone 4.0 × 7 mm implant in the inter-radicular septum.

Upon completion of the preparation, the surgical site presented intact bone walls and a shape suitable for implant placement. An Implacil Duecone implant (4.0 × 7 mm) was slowly placed, positioned approximately 1 mm below the level of the bone septum, achieving a final torque of 25 Ncm, measured by the manual torque wrench, which indicated satisfactory primary stability (Figure 7). Subsequently, the cover screw was placed. This implant has an internal conical connection of the Morse taper type.

**Figure 8:**
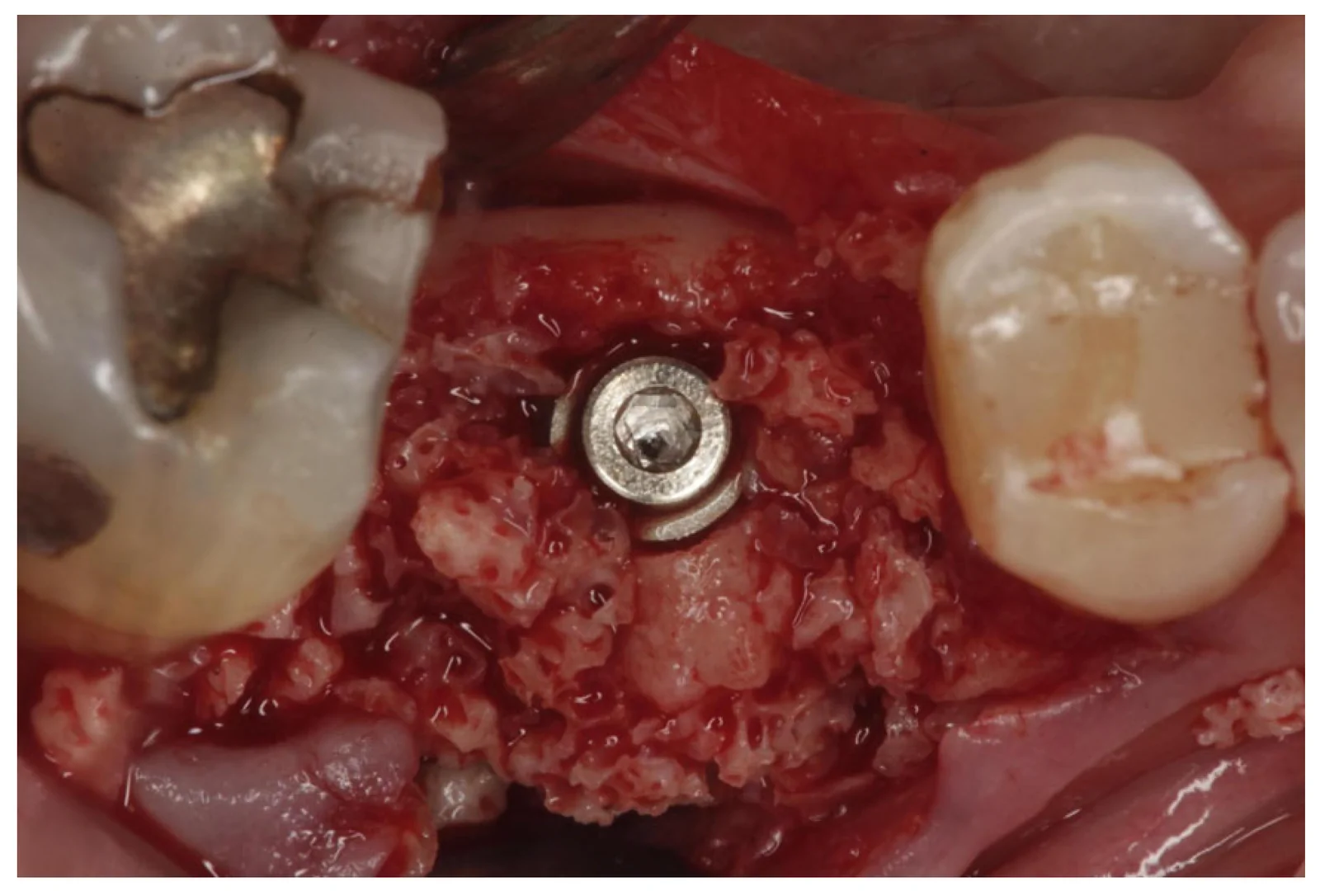
Filling the socket with xenogeneic biomaterial for socket preservation after implant placement.

**Figure 9:**
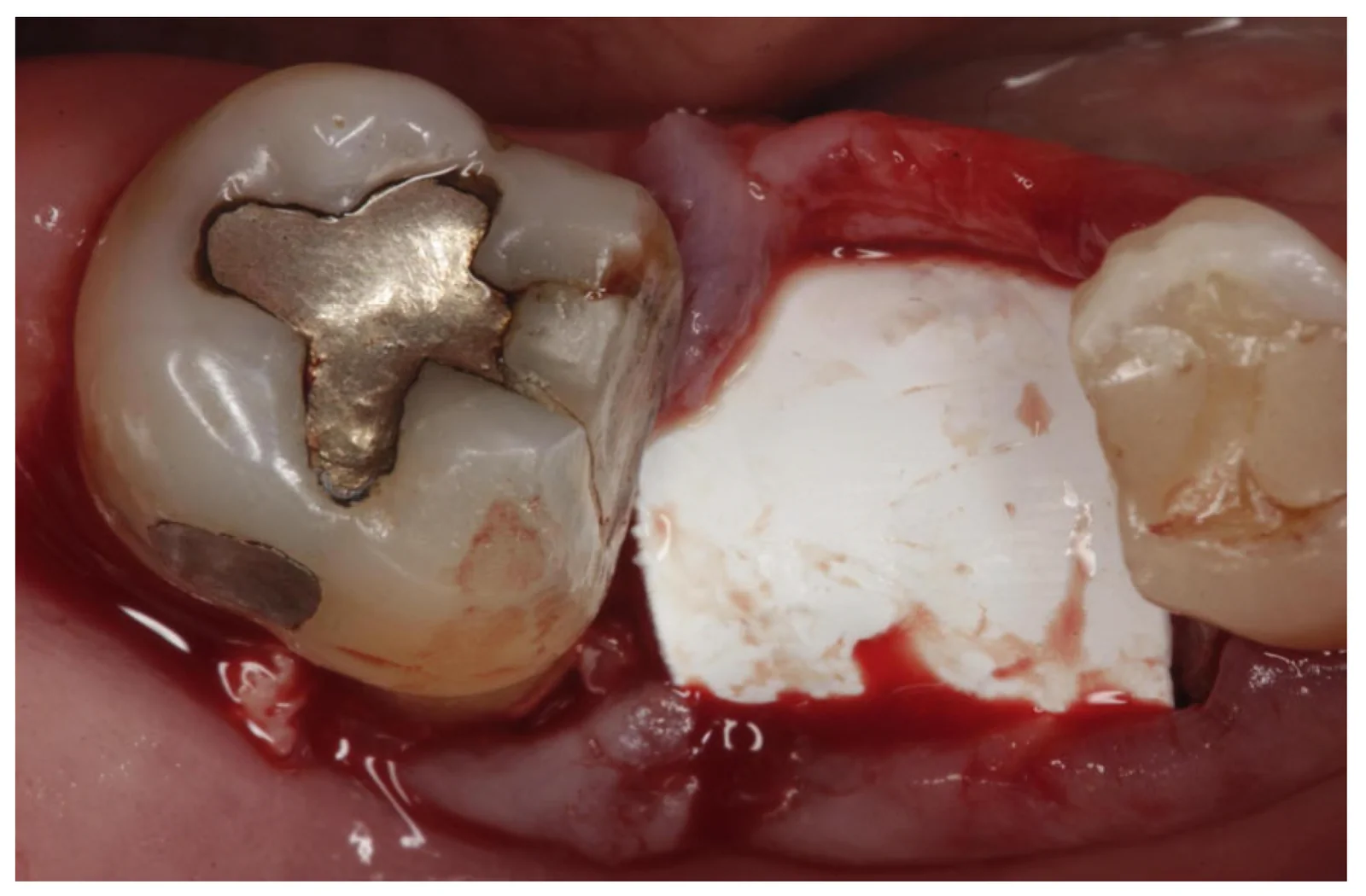
Dense PTFE barrier positioned over the socket for sealing and cell exclusion.

**Figure 10:**
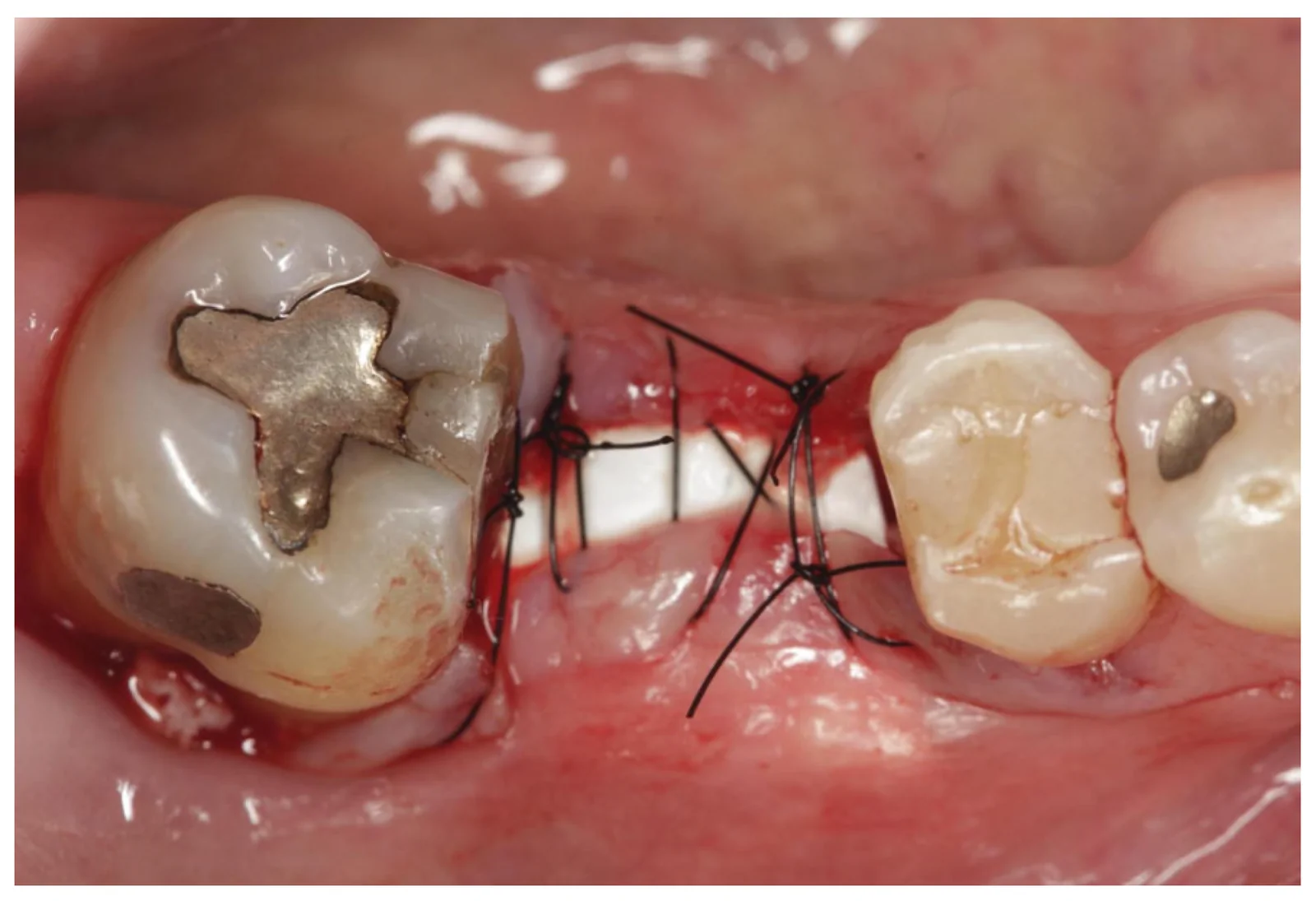
Final appearance of the surgical procedure after suturing with 5-0 nylon thread.

For socket preservation, the residual socket space was filled with Bonefill Large (Bionnovation) xenogeneic biomaterial (Figure 8). Subsequently, the area was covered with a dense polytetrafluoroethylene (d-PTFE) barrier (Criteria) to seal the socket and exclude soft tissue cells (Figure 9). Simple interrupted sutures using 5-0 nylon threads were placed (Figure 10).

Sutures were removed after 14 days, while the barrier was removed after 28 days. The healing period proceeded without clinical complications. After four months, the implant was uncovered; adequate new bone formation was observed around the platform, along with conditions favorable for prosthetic rehabilitation. An Ideale abutment (Implacil, São Paulo, Brazil), dimensions 4.5 × 4 × 1.5, was selected and screwed into the implant with 30 Ncm, as recommended by the manufacturer. A metal–ceramic crown was fabricated and cemented, restoring the masticatory function and esthetics of the lost tooth. The choice of a cement-retained restoration was because of the short inter-occlusal space required for a screw-retained restoration and the space required for resin above the screw.

**Figure 11:**
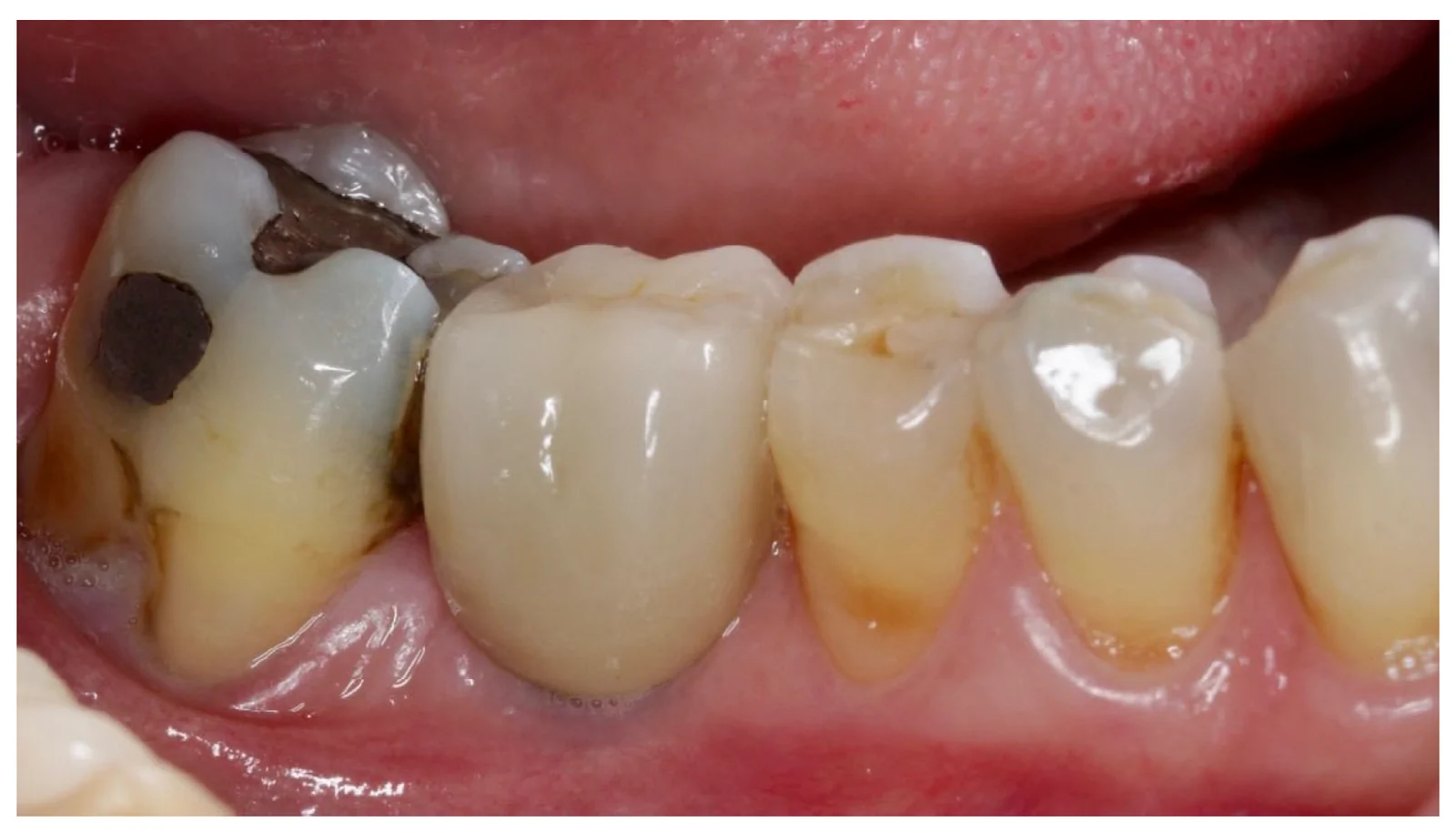
Clinical aspect at 4-year follow-up.

**Figure 12:**
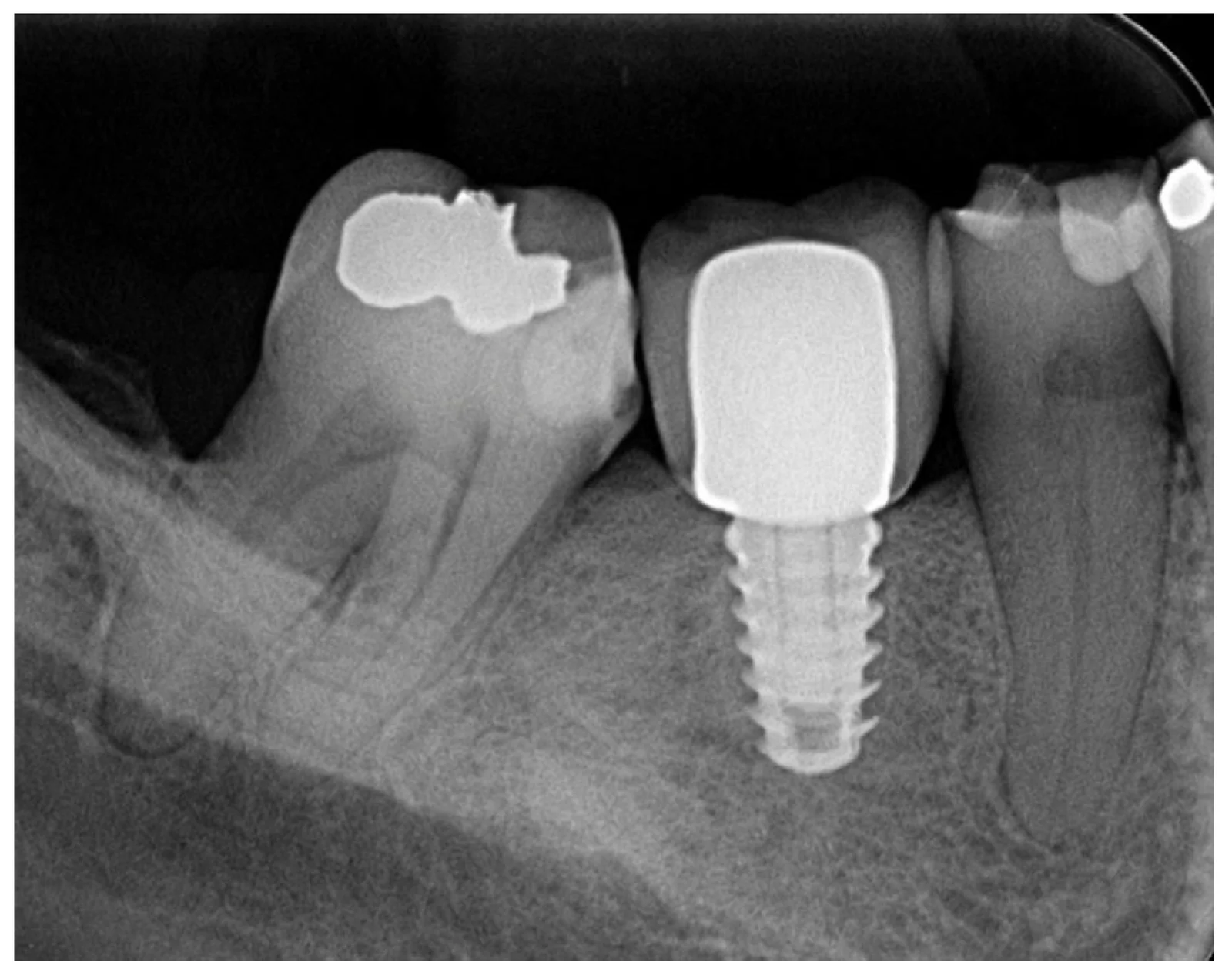
Periapical radiograph at 4-year follow-up.

The patient returned for a routine check-up and to treat another tooth through root canal treatment only four years after implant treatment completion; the peri-implant tissues were found to be healthy, despite the presence of only a narrow band of keratinized mucosa on the buccal aspect of tooth 46 (Figure 11 and Figure 12). Because a digital periapical radiograph was not taken at the time of implant placement, we could not measure adequate marginal bone loss, and just suppose upon visualization of the control x-ray that minimum bone loss occurred.

## 3. Discussion

The case presented represents a challenging clinical scenario frequently described in the posterior mandible, characterized by low trabecular bone density, a wide post-extraction socket, and limited apical bone for implant anchorage. These factors are widely recognized as negative determinants for achieving primary stability in immediate implants, especially in mandibular molars, where treatment predictability may be reduced [1,4].

In such situations, the literature identifies the inter-radicular septum as the most favorable region for immediate implant placement, regarding both three-dimensional positioning and long-term survival rates [3,4,16]. In the present case, careful preservation of the septum during tooth extraction and its use as the primary anchorage site enabled the execution of the immediate protocol despite the loss of the buccal bone plate, reinforcing the importance of this anatomical structure in molar sockets.

Primary stability is considered a key determinant of success for immediate implants, as excessive micromotion during the initial healing phase can compromise the osseointegration process [1,2,5]. Clinical studies demonstrate that insufficient initial stability values are associated with fibrous tissue formation at the implant–bone interface, particularly in low-density bone^2^. In the reported case, the final insertion torque of 25 N/cm indicates satisfactory primary stability—a value consistent with those described in the literature as adequate for immediate implants in posterior regions [6,9,14].

Osseodensification has been widely investigated as an alternative to conventional osteotomy preparation, especially in sites with low bone density [3,7,8]. Unlike traditional drilling, this technique promotes the lateral compaction of autogenous bone, an increase in peri-implant bone density, and controlled expansion of the inter-radicular septum. These biomechanical effects were clinically observed in the present case, where densifying instrumentation resulted in a surgical site with intact bone walls and a shape compatible with the planned implant. A recent study compared molar septum instrumentation with osseodensification or Piezoelectric implant site preparation (PISP), with the osseodensification group showing slight higher values in terms of primary and secondary stability, measured by ISQ, but with no statistical differences. Also the site preparation time demanded was faster with osseodensification than with PISP, but the insertion torques were lower than in the PISP group [17].

Experimental studies and clinical trials demonstrate that osseodensification is associated with higher insertion torque and implant stability quotient values during both the primary and secondary phases [6,9,11,14]. Furthermore, the compacted bone fragments generated during drilling act as nucleating agents, promoting osteogenesis and contributing to stability maintenance throughout the healing process [11,12]. This mechanism helps explain the favorable clinical outcome observed in this case, even in a site with unfavorable anatomical characteristics.

Another relevant aspect concerns the elastic recoil effect of bone subjected to osseodensification, described as a factor associated with improved secondary stability [7,12,14]. This behavior promotes continuous compression of the implant by the densified bone walls, fostering greater implant–bone contact during the remodeling phase. In the present report, the absence of postoperative complications and the adequate new bone formation observed upon re-entry corroborate these findings.

The macro-geometry of the implant used also warrants consideration. Implants with a tapered design have been associated with better initial stability in low-density bone, especially when combined with techniques that increase lateral friction, such as osseodensification [6,9,13]. The combination of the Implacil Duecone implant and the densifying preparation appears to have contributed positively to the primary stability achieved in this clinical case [13]. Unfortunately, at the time of implant placement no Resonance Frequency Analysis devices were available for us to measure the implant stability quotient, so we just had the installation torque as initial data, which was measured by the manual wrench.

Clinical studies, comparative analyses, and retrospective reviews report high success rates, long-term stability maintenance, and reduced marginal bone loss for implants placed using osseodensification compared to conventional protocols, including in immediate implant placement and posterior regions [10,13,15,16,17,18,19,20]. The results observed in this report align with this evidence, reinforcing the technique’s predictability in complex clinical situations. More controlled clinical studies are needed before extrapolating the result of this single case report to routine practice.

In this case we chose to fill the gaps with a xenograft bone substitute and to seal the alveolus with a dense PTFE barrier. As such, we did not need to achieve primary closure, which would lead us to do vertical incisions and probably increase morbidity in the area and discomfort for the patient. Previous studies of our group have already shown that the use of a d-PTFE barrier and the filling of the alveoli with xenografts can lead to great ridge preservation, either with or without immediate implant placement [21,22]. Other clinical studies have shown similar outcomes, either with the use of a d-PTFE barrier or through gap filling with xenografts for alveolar ridge preservation [23,24].

As a limitation of this case report, we point out the lack of parameters for marginal bone loss around the implant, as there is no soft tissue measurement data (probing the depth or band of keratinized tissue). Because the patient presented during the COVID-19 pandemic, some of the procedures were performed aiming to just solve the patient’s problem, and so we did not think in terms of standard procedures or data acquisition. Also the patient waited 4 years to return to the clinic for another treatment, leaving us without a proper follow-up for the case. We believe that with soft tissue augmentation the case would have finished in a better way, likely with more stability in a long-term.

## 4. Conclusions

Within the limitations of this case report, it can be concluded that the osseodensification technique enabled immediate implant placement in a mandibular molar site by inter-radicular septum expansion. This allowed for satisfactory primary stability and ensured conditions for the osseointegration process in this case.

## Data Availability

The original data presented in the study are included in the article; further inquiries can be directed to the corresponding author.

## Abbreviations

The following abbreviation is used in this manuscript:

PTFE	Polytetrafluorethylene
